# Trichotomy of awareness, outlook and practice of food handlers towards food and water safety in food establishments in Addis Ababa, Ethiopia

**DOI:** 10.3934/publichealth.2020021

**Published:** 2020-05-06

**Authors:** Aderajew Mekonnen Girmay, Sirak Robele Gari, Azage Gebreyohannes Gebremariam, Bezatu Mengistie Alemu, Martin R. Evans

**Affiliations:** 1 Ethiopian Institute of Water Resources, Addis Ababa University, Addis Ababa, Ethiopia; 2 College of Health and Medical Sciences, Haramaya University, Haramaya, Ethiopia; 3 Microbiology Consultant and Laboratory Director, New York, USA

**Keywords:** awareness, outlook, practice, food handler, Addis Ababa

## Abstract

**Introduction:**

Food and waterborne diseases are the commonest global public health problems. Specifically, in Ethiopia, public health problems associated with deterioration of food and water safety situations are much more complicated due to poverty, economic and environment related risks. Awareness, outlook and practice of food handlers are the three important factors that play major roles in the occurrence and outbreak of food borne diseases. Therefore, this study aimed to evaluate awareness, outlook and practice of food handlers towards food and water safety.

**Methods:**

Institution based cross sectional study was conducted among food handlers of Addis Ababa city administration. In this study, 416 participants were selected using a stratified, simple random sampling technique; and were interviewed using structured questionnaire. Linear Regression Model and analysis of variance were used for data analysis.

**Results:**

In this study, 55.5%, 66.1% and 60.6% of the food handlers had good awareness, outlook and proper hygiene practices respectively. This study revealed that, 17.5% and 23.1% of the respondents did not know about food and water borne disease respectively. Only 39.4% of the participants had proper practice of covering mouth with tidy cloth when they cough. Moreover, 75.7% of the food handlers reported that they did not wear personal protective devices during the working time. Predictor variables like educational status and length of work experience were correlated positively and significantly with awareness. However, being married was correlated negatively with awareness.

**Conclusion:**

Assessing awareness, outlook and practice of food handlers regarding food and water safety is a vital activity to reduce public health problems. Significant number of food handlers had poor awareness, outlook and practice towards food and water safety. There is a call for enhancing the awareness, outlook and practice of food and water safety to achieve an excellent practice. Better food and water safety policy and firm regulatory actions are needed.

## 1. Introduction

Globally, deterioration of food and water safety practices have become major public health concern due to the spread of food and water borne diseases [1]. At the global scale, food and water associated infectious diseases are significantly correlated with socio-environmental factors, impacting all regions [2],[3]. The issue of food and water safety is much more complicated in developing countries due to several reasons [4]. Poor socioeconomic status is one of the leading causes of consumption of unsafe food that contributed to outbreaks of infectious diseases in communities [5],[6]. Globally, about 88% of diarrhea-associated deaths are attributed to unsafe food and water consumption [7],[8]. Moreover, two million annual deaths and four billion cases of diarrhea are attributed to food and water-borne diseases [9]. A study done in Nigeria reported that, according to 33% of the respondents, restaurant food commonly caused food borne illness [10]. Furthermore, there is a linkage between food establishments and approximately 60% of food borne disease outbreaks [11]. There are plenty of conditions where food and water borne disease affect the health of people across the globe [12],[13]. Further, the chances of food and water contamination mostly depend on the health status of food handlers and their hygiene behaviors, awareness and practices [14],[15]. The trichotomy of awareness, outlook and practice of food handlers are the three important factors that play vital roles in the incidence and outbreak of food and water borne diseases [16]. People involved in food handling and having poor personal hygiene and lacking awareness of ways to preventing food and water borne diseases, could be potential sources of infections [17]. Though food handlers are expected to maintain a high degree of personal hygiene and careful handling of food; they have inadequate perception about how food and water could be contaminated, and usually have low standards of personal hygiene for the tasks they are expected to perform [18]. Moreover, poor food handling practices among food handlers is common [19]. Due to poor hygienic practice and the nature of their work, food handlers can transmit a variety of food and water borne diseases to their customers [17],[20]. Food borne illnesses outbreaks can be caused by poor hygienic practices of food handlers in conjunction with poor sanitary conditions of food outlets [21].

Even though food and water safety packages have been implemented in Addis Ababa, there are several health problems mainly recurrent food and water borne outbreaks [22]. According to the 2016 Addis Ababa health bureau report, there is high prevalence of food borne disease in Addis Ababa though its sources are not well known and studied in depth. According to the 2017 report by Addis Ababa Food, Medicine and Health Care Administration and Control Authority (AAFMHACA), food establishments located in Addis Ababa are suspected to be key sources of food borne outbreaks which might arise from poor awareness, outlook and practice of food handling personnel, poor quality of drinking water, poor waste water and solid waste management, lack of wash facilities and poor water storage conditions. Food borne disease is an important public health problem, causing morbidity and mortality. However, except for verbal reports, no studies have been conducted on the awareness, outlook and practice of food handlers. Because of this, a significant number of customers of the food establishments have been exposed to different gastro-intestinal problems. Therefore, this study has an important contribution to solving community health problems resulting from poor awareness, outlook and hygiene practice of food handlers.

## 2. Methods

### 2.1. Operational definitions

Good Awareness: To assess the level of awareness, respondents were asked 13 questions from the questionnaire and those who scored greater than or equal to the mean value were considered as having good awareness and those who scored less than the mean value were considered as having poor awareness [5]. Good outlook: To assess the level of outlook, respondents were asked 6 questions from the questionnaire and those who scored greater than or equal to the mean value were considered as having good outlook and those who scored less than the mean value were considered as having poor outlook. Good Practices: To assess the level of practices, respondents were asked 11 questions from the questionnaire and those who scored greater than or equal to the mean value were considered as having good practices and those who scored less than the mean value were considered as having poor practices [5].

Food establishments: Institutions that provide food and drinks for selling to customers.

Food handler: A person who prepares and handles food in food establishments.

Big food establishment: Hotels with one or more stars.

Small food establishments: Food and drink vender institutions that are small (non-star hotels, bar and restaurants, cafe and restaurants, restaurants etc.).

Slum area: Area with relatively poor sanitation and hygiene practices.

Non-slum area: Area with relatively good sanitation and hygiene.

### 2.2. Description of the study area

**Figure 1.**
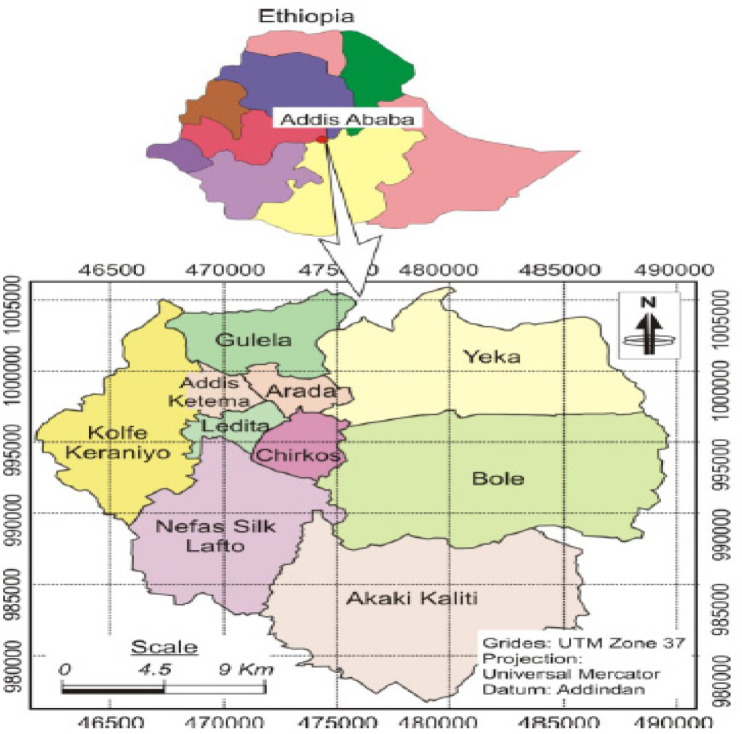
Map of Addis Ababa city administration [23].

The study was conducted in Addis Ababa city located in Upper Awash River basin, the capital of the Federal Government of Ethiopia and where the African Union Headquarters are housed. According to the 2017 AAFMHACA report, there are 1141 licensed food establishments, employing 4565 food handlers. Of the total licensed food establishments, 95 (8%) are large (hotels with one or more stars) and the remaining 1046 (92%) are small food establishments which include unranked (non-star hotels, bars, restaurants, cafes etc.) [23]. The location map of Addis Ababa city is depicted below in Figure 1.

### 2.3. Study design

An institutional based cross sectional study was conducted among food handlers of Addis Ababa city administration from June to July 2019.

### 2.4. Study population

All selected food handlers located in Addis Ababa city administration.

### 2.5. Inclusion and exclusion criteria

All food handlers aged 18 years and above were the inclusion criteria. However, casual food handlers were excluded.

### 2.6. Sample size determination

The sample size was calculated using a single population proportion formula (EPI INFO version 7.2.2.6). With the assumption of 95% confidence interval, tolerable margin of error (d) 5%, and the previous population proportion of good outlook (P = 46.5% ) among food handlers [17]. Then, the sample size (n) was calculated as follows:

$$n=\frac{\left(Z^{2}\right)*P\left(1-P\right)}{d^{2}}$$ (1)

where, n = the calculated sample size, z = standard score corresponding to 95% CI, P = the assumed proportion of good outlook of food handlers (46.5%) [17] and d = marginal error (5%).

Then, $\text{n}=\frac{\left(1.96\right)\times \left(1.96\right)\times \left(0.465\right)\times \left(1-0.465\right)}{0.05\times 0.05}=382$.

Adding 10% non-response rate, the sample size was = 420.

### 2.7. Sampling procedure

**Figure 2.**
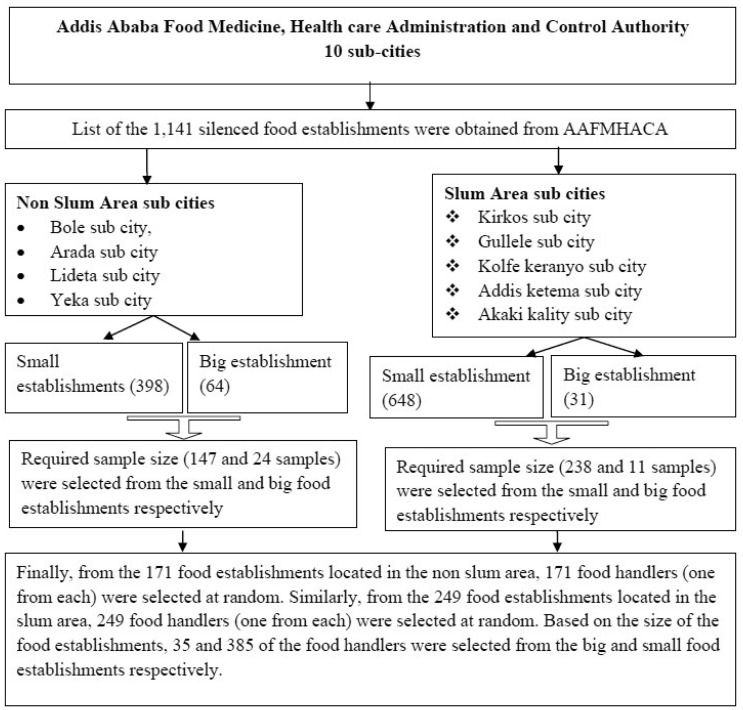
Systematic structure of sampling procedure of the study.

The study participants were selected using a stratified, simple random sampling technique. To collect data, a listing of the 1141 licensed food establishments was obtained from AAFMHACA. These 1141 existing food establishments were stratified in to slum and non-slum area based on their location. Similarly, the food handlers were stratified in to two based on their work location (slum and non-slum area). Further, food handlers working in big and small food establishments were stratified in to two. Based on this, sample allocation was done to the slum and non-sum area in addition to the big and small food establishments. Then, after the food handlers were stratified based on their work location and size of food establishments (big or small), one food handler from one food establishment was selected at random. Based on the sample allocation, 171 and 249 food handlers were taken from the food establishments located in the non-slum and slum area respectively. From the non-slum area (171), 24 samples from the big food establishments and 147 samples from the small food establishments were taken. Besides, from the slum area (249), 11 samples from the big food establishments and 238 samples from the small food establishments were taken. Further, based on the sample allocation of the size of the food establishments, from the slum and non-slum area a total of 35 and 385 samples of food handlers were taken from the big and small food establishments respectively. Lastly, using a simple random sampling technique, 420 food handlers were selected to assess awareness, outlook and practice of food handlers towards food and water safety. A stratified random sampling technique was conducted, in both slum and non-slum area and the size of food establishments of Addis Ababa. The main purpose of stratification was for the sake of representativeness of food handlers. In summary, the sampling procedure for this study is depicted below in Figure 2.

### 2.8. Data collection procedures

Data enumerators were identified based on professional capability and technical experience in collecting the required data. Accordingly, fifteen health professionals with Bachelor of Science with extensive experience in a similar data collection practices were employed. In addition, four Masters' degree holders acted as supervisors were assigned. Two-days training were given to the data collectors and supervisors. To collect data, first the data collectors informed the food establishment managers about the aim of this study and reached agreements. Then, to select the food handlers, list and address of food handlers were taken from the manager. Next to this, arrangement was done for meetings between the randomly selected food handlers and the trained data collectors considering the convenient time and place for the food handlers. The data collectors did not report to managers as they were meet the food handlers. Finally, after written consent was obtained from each study subjects, the data was collected from food handlers through face-to-face interview using structured questionnaire.

### 2.9. Data quality assurance

A questionnaire was prepared in English and translated in to Amharic and back to English to keep the consistency of questions. The quality of data was ensured through training of data collectors, close supervision, prompt feedback and daily recheck of completed questionnaire.

### 2.10. Data analysis

All data were checked for correctness of information and code. Data analyses were performed by using SPSS (Statistical Package for the Social Sciences) software version 20. Descriptive statistics such as frequency (%) for categorical and mean and standard deviation for numerical data were used to sum up the data. Moreover, Linear Regression Model and analysis of variance (ANOVA) with 95% confidence level were used. In all analysis, P-value less than 0.05 were considered statistically significant. A questionnaire consisting of 13 questions was prepared to assess awareness of food handlers. Each question had at least two choices. A correct answer was given a score of 1, whereas a score of 0 was given for a wrong or incorrect answer. Finally, all individual answers were summed up for total scores and calculated for mean. Further, to assess outlook of food handlers, there were six (6) questions in this part and the food handlers were interviewed regarding their awareness level towards food and water safety. Each question had at least two choices. A correct answer was given a score of 1, whereas a score of 0 was given for a wrong or incorrect answer. All individual answers were summed up for total scores and calculated for mean. To assess practice of food handlers, a questionnaire consisting of 11 questions was prepared. Each question had at least two choices. A correct answer was given a score of 1, whereas a score of 0 was given for a wrong or incorrect answer. All individual answers were summed up for total scores and calculated for mean.

### 2.11. Study variables

A: independent or explanatory variables:

The predictor variables of this study were sex, age, marital status, religion, educational status, length of work experience of the food handlers.

B: dependent or outcome or response variables:

Outcome of this study were awareness, outlook and practice of food handlers.

### 2.12. Ethical consideration

Ethical approval was obtained from Ethiopian Public Health institute scientific and ethical review board Ethiopia with the reference number EPHI 613/138 in June 2019. To collect the data, written consent was obtained from each respondent. They were informed that their participation was voluntary. Confidentiality and privacy of respondents were ensured throughout the research process. The study design does not harm those taking part and it does not include any identifying information like name, or address of a respondents on questionnaire. Then, data was collected after assuring the confidential nature of responses.

## 3. Results

### 3.1. Socio-demographic characteristics of the food handlers

Out of the 420 food handlers 416 consented to this study providing 99.1% response rate. There were 76.9% female and 23.1% male respondents. Above half of the respondents were in the age group of 18–22 years old (51.7%) followed by the age group of greater than 27 years old (25%). The mean age of the respondents was 24.93 years. More than three-fourth or 76% of the food handlers had ability of at least reading and writing while 24% of them were illiterate. Nearly all (93%) of the food handlers were single and 5% of them were married. Regarding religion, most of the participants (82.5%) were Orthodox Christians followed by Muslim (11.1%) and other religions (6.5%). The average length of food handlers work experience was found to be 2.46 years (Table 1).

**Table 1.** Socio-demographic characteristics of the food handlers (n = 416).

Study variables	Category/Item	Frequency	Percent (%)
Sex of the food handlers	Male	96	23.1
	Female	320	76.9
Age group of the food handlers	18–22 years	215	51.7
	23–27 years	97	23.3
	>27 years	104	25.0
Educational status of the food handlers	Illiterate	100	24.0
	At least read and write	316	76.0
Marital status of the food handlers	Single	387	93.0
	Married	21	5.0
	Divorced and others	8	1.9
Religion of the food handlers	Orthodox	343	82.5
	Muslim	46	11.1
	Others	27	6.5
Work experience of the food handlers	Above three month and below one year	205	49.3
	Between 1–3 years	82	19.7
	Above 3 years	129	31.0
Mean and standard deviation of the age of the respondents		24.93 ± 7.59	
Mean and standard deviation of the work experience of the respondents		2.46 ± 3.02	

### 3.2. Awareness of food handlers on food and drinking water safety

The percentages mean score of awareness questions was found to be 78.18. However, based on the cutoff point, only 55.5% of the food handlers had good awareness. In this study, 17.5% and 23.1% of respondents did not know about food and water borne disease respectively. Majority of food-handlers (86.8%) knew contaminated food and water causes public health problem and 83.7% of them had awareness about water contamination if it is not handled properly. Further, nearly 70% of the food handlers had awareness about acute watery diarrhea. In addition, 63.5% and 62.3% of the food handlers knew about Salmonella, hepatitis “A” virus as food and water borne pathogens respectively; and 62% of them knew that bloody diarrhea is transmitted through food and water borne pathogens. Further, 73.1% of the food handlers had awareness of typhoid fever as it is transmitted by contaminated food and water. Of the total respondents, 66.6% of them knew food and water could be easily contaminated at the point of use. Almost all of the respondents (95.7%) and 95% knew mode of food and water borne diseases transmission and prevention mechanisms respectively (Table 2).

**Table 2.** Awareness of food handlers on food and water safety (n = 416).

No	Questions or study variables	Answers% (n)	
		Correct awareness	Incorrect awareness
1.	Do you know about food borne diseases?	82.5 (343)	17.5 (73)
2.	Do you know about water borne diseases?	76.9 (320)	23.1 (96)
3.	Do you know contaminated food and water causes public health problem?	86.8 (361)	13.2 (55)
4.	Do you know water can be contaminated if it is not handling properly?	83.7 (348)	16.3 (68)
5.	Do you know about Acute watery diarrhea (AWD)?	69.5 (289)	30.5 (127)
6.	Do you know Salmonella is a food and water borne pathogen?	63.5 (264)	36.5 (152)
7.	Do you know hepatitis “A” virus is a food borne pathogen?	62.3 (259)	37.7 (157)
8.	Do you know bloody diarrhea is transmitted by contaminated food and water?	62.0 (258)	38.0 (158)
9.	Do you know typhoid fever is transmitted by contaminated food and water?	73.1 (304)	26.9 (112)
10.	Do you know food and water can be easily contaminated at the point of use or at food establishment?	66.6 (277)	33.4 (139)
11.	What is the source of your awareness about food and water borne diseases? Health professionals…1; Radio and television…2; Formal training…3; Posters…4; Sanitary inspectors…5; Absence of training….6	99.0 (412)	1 (4)
12.	What is the mode of transmission of food and water borne diseases? Through contaminated water…1; Through contaminated food…2; Through contaminated hand…3; Through utilizing improved toilet...4; Dirty work environment…5	95.7 (398)	4.3 (18)
13.	What is the prevention mechanism of food and water borne diseases? Washing hand with soap and safe water before meal………1 Washing hand with soap and safe water after defecation ….2 Isolate sick food handler until he/she treated……………….3 Properly cooking food and safe water handling……………4 Habit of eating raw beef and vegetables………..6	95 (395)	5 (21)
	Total percentage mean score of correct answer to awareness	78.18 ± 22.02	

### 3.3. Outlook of food handlers on food and drinking water safety

The result of the study indicated that, the percentages mean score of outlook questions was 72.88. However, depending on the cutoff point, only 66.1% of the food handlers had good outlook. Of the total participants, 71.9% of the food handlers had correct outlook about using protective tidy clothes that it minimizes contamination of food and water. Further, 76.9% and 78.4% of the food handlers believed that, taking treatment is mandatory when food handler is sick and washing hands should be obligatory before handling cooked food and drinking water respectively. Moreover, 27.9%, 33.9% and 28.1% of the food handlers did not believe that raw food should be separated from cooked food, treated water cannot be easily contaminated if not properly stored at safe container and food and water borne diseases can not arise from food establishments respectively (Table 3).

**Table 3.** Outlook of food handlers on food and drinking water safety (n = 416).

Sr.no	Questions or study variables	Answers% (n)	
		Correct outlook	Incorrect outlook
1.	Do you believe using protective tidy clothes minimizes both for food and water contamination at food establishment?	71.9 (299)	28.1 (117)
2.	Do you believe taking treatment is mandatory when food handler sick?	76.9 (320)	23.1 (96)
3.	Do you believe washing hands should be obligatory before holding food and touching drinking water?	78.4 (326)	21.6 (90)
4.	Do you believe raw food should be separated from cooked food?	72.1 (300)	27.9 (116)
5.	Do you believe treated water can be easily contaminated if not properly stored at safe container?	66.1 (275)	33.9 (141)
6.	Do you believe food and water borne diseases can be arising from food establishments?	71.9 (299)	28.1 (117)
	Total percentage mean of correct answer to outlook	72.88 ± 35.50	

### 3.4. Practice of food handlers on food and drinking water safety

The study result revealed that, the percentages mean score of practice questions was 67.48. But, based on the cutoff point, only 60.6% of the food handlers had good or proper hygiene practices. Of the total participants, 67.5% washed drinking water containers with sanitizers regularly. The action of putting food and drinking water in clean containers had the highest score (91.3%) followed by regular washing of drinking water glass (89.9%) with sanitizers. Further, 79.8% of the food handlers had a practice of washing hands with soap and clean water before meal and after defecation. Out of the total participants, 75.5% of food handlers had a practice of washing food utensils with sanitizers and disinfectants before serving with it. In addition, 71.2% of them used tidy clothes for cleaning food utensils regularly. Of the total respondents, 82% washed their hands with soap and clean water before holding cooked foods. In our finding, only 39.4% of the food handlers had proper practice of covering mouth with tidy cloth when they cough, whereas 32.79% cut their nail when it becomes tall. Moreover, 75.7% of the food handlers reported that they did not wear personal protective devices like white gown and gloves during the working time (Table 4).

**Table 4.** Practice of food handlers on food and drinking water safety (n = 416).

Sr.no	Questions or study variables	Answers% (n)	
		Proper practice	Improper practice
1.	Do you wash drinking water containers with sanitizing and disinfectants regularly?	67.5 (281)	32.5 (135)
2.	Do you wash drinking water glass with sanitizing and disinfectants after every customer use it regularly?	89.9 (374)	10.1 (42)
3.	Do you wash your hands with soap and clean water before meal and after defecation?	79.8 (332)	20.2 (84)
4.	Do you wash food utensils with sanitizing and disinfectants before serving with it?	75.5 (314)	24.5 (102)
5.	Do you use tidy clothes for cleaning food utensils regularly?	71.2 (296)	28.8(120)
6.	Do you wash your hands with soap and clean water before holding cooked food?	82.0 (341)	18.0(75)
7.	Do you put food and drinking water in clean containers?	91.3 (380)	8.7 (36)
8.	Do you cook food thoroughly before ready for consumption?	88.7 (369)	11.3 (47)
9.	Do you cover your mouth with tidy clothe while you coughing?	39.4 (164)	60.6 (252)
10.	Do you cut your nail when it becomes tall (short nail at the time of interview?	32.79 (136)	67.3 (280)
11.	Do you wear at least white gown or made glove during the work days?	24.3 (101)	75.7 (315)
	Total percentage mean percentage of correct answer to practice	67.48 ± 22.65	

### 3.5. Linear Regression Analyses

In the Linear Regression analysis of this study, five predictor variables were identified to test their impact on the obtained results. Moreover, one additional model (mean score awareness of food handlers of this study) was testing its impact on food and water safety practice and outlook (Tables 5–7).

### 3.6. Association between awareness mean score and explanatory variables

As shown below in Table 5, associations between awareness mean score and sex, age, educational status, marital status and work experience of food handlers exists. Except sex and age, all the independent (educational status, marital status/being married and work experience of food handlers) variables had significant relationship (P-value < 0.02) with the dependent variable (awareness). The predictor variables (Educational status and length of work experience) of the food handlers had a significant positive correlation with awareness. Beta (β) is significant (P = 0.00) for the first predictor and (P = 0.02) for the second predictor. However, being married was significantly negatively correlated with awareness. Beta (β) is significant (P = 0.00) for being married. This indicated that, the effect of increased educational status had increased the awareness of the respondents on average by 0.39 or by 3.9%. Similarly, the expected awareness of food handles increased on the average by 0.11 or by 1.1% due to increased length of work experience as food handler. However, being married decreased the awareness of the food handlers by 0.16 or by 1.6% (Table 5).

**Table 5.** Association of awareness mean score with the predictable variables using Linear Regression Model (n = 416).

Predictor/independent variables	Percentage of Awareness mean score of the food handlers							
	Standardized Coefficient (Beta)	F	Df	Std. error	T	P-value	95% Confidence Interval (CI)	
							Lower Bound	Upper Bound
Sex	0.02	0.13	1	0.04	0.353	0.72	−4.137	5.95
Age	−0.04	0.57	1	0.14	−0.75	0.45	−0.39	0.17
Education	0.39	76.16	1	2.33	8.73	0.00	15.72	24.86
Marital status	−0.16	10.30	1	3.09	−3.21	0.00	−15.98	−3.84
Length of work experience	0.11	5.12	1	0.36	2.26	0.02	0.11	1.50

### 3.7. Effect of food and water safety awareness on food and water safety practice

The result of the study showed that food and water safety awareness had a significant effect on food and water safety practices. The linear regression analysis established that food and water safety awareness could statistically predict food and water safety practices (F = 183.53; df = 1; P = 0.00) with Standardized Coefficient (Beta) = 0.55 at 95% CI (Table 6).

**Table 6.** Effect of awareness on food and water safety practice using a Linear Regression Model (n = 416).

Predictor/independent variables	Practice							
	Standardized Coefficient (Beta)	F	df	Std. error	T	P-value	95% CI	
							Lower Bound	Upper Bound
Awareness mean score	0.55	183.53	1	0.04	13.55	0.00	0.49	0.65

### 3.8. Effect of food and water safety awareness on food and water safety outlook

The finding of the study revealed that, food and water safety awareness had a significant effect on food and water safety outlook. The linear regression analysis indicated that, food and water safety awareness could statistically predict food and water safety outlook (F = 252.5; df = 1; P = 0.00) with Standardized Coefficient (Beta) = 0.62 at 95% CI (Table 7).

**Table 7.** Effect of awareness on food and water safety outlook using a Linear Regression Model (n = 416).

Predictor/independent variables	Outlook							
	Standardized Coefficient (Beta)	F	Df	Std. error	T	P-value	95% CI	
							Lower Bound	Upper Bound
Awareness mean score	0.62	252.49	1	0.06	15.89	0.00	0.87	1.12

## 4. Discussion

In this study, 17.5% and 23.1% of respondents did not know about food and water borne diseases respectively. The higher lack of awareness about food and water borne diseases could be due to the lack of formal and informal training. Moreover, most of the food establishment managers and owners of food establishments hired food handlers who come from rural part of the country where they did not have any experience and awareness regarding food and water borne disease. The finding of the study revealed that, 27.9% of the food handlers did not consider separation of raw food and cooked food important and 33.9% thought that treated water cannot be easily contaminated at the food establishment level. This indicated that, there is poor outlook on the causes of cross contamination which could arise due to keeping cooked and raw food together. As a result of this, many customers can be exposed to different health problems. Moreover, even they believe the drinking water comes from secured and treated water supply, it can be a source of many water borne diseases if it is not stored properly in clean places. The study revealed that, only 39.4% and 32.79% of the food handlers had proper practice of covering mouth with tidy cloth while coughing and cut their nail when it becomes tall respectively. This revealed that, most of the food handlers did not have good practice to reduce communicable disease that can be transferred through droplets and sneezing. As a result of this, many customers of food outlets might be exposed to different health problems. Moreover, their tall nail can accumulate various diseases causing agents and can be a reason of disease outbreaks among them and their customers. In addition, 75.7% of the food handlers reported that they did not wear personal protective devices like white gown and gloves during the working time. This indicated that, there was negligence to hygiene and sanitation practice both by the food handlers and the owners of the outlets.

In this study, 78.2% of the food handlers had a mean percentage score of awareness on food and water safety. This was consistent with the study done in Nigerian food handlers [24]. This might be due to similarity of economic status and educational provision. However, the finding was lower than a study done in Salvador, Brazil [25]. This can be due to differences in development and attention given to food and water safety. The World Health Organization recommends that food handlers should have enough awareness to protect food from all kinds of contamination [26]. However, the study result indicated that, only 55.5% of the respondents had good awareness. This could be due to lack of formal training and strong attention on food and water borne diseases by the concerned bodies. The finding of the study indicated that, the level of food handlers' percentage mean score of practices was 67.48%. This was higher when compared to a study done in Malaysia [27]. Furthermore, in our finding, 60.6% of the food handlers had proper practice. However, this result was lower than the study done in Brazil [28]. This could be due to low attention of Government and other stakeholders in creating strong regulatory body and awareness among the food handlers in Addis Ababa. Moreover, the second probable reasons for the differences might be due to differences in socio-demographic status and environmental factors.

The result of the study revealed that, of the explanatory variables sex and age had not significant association with awareness. This indicates that, if once a person is an adult, age might not be important influencing factor on awareness. Because once a person reaches adulthood, the subsequent awareness acquiring solely depends on formal and non-formal education, experience and other personal efforts. On the contrary, others claimed presence of significant associations (P < 0.05) between age and awareness of food safety [29]. This result needs future research to obtain additional evidence.

In our study, educational status of food handlers was significantly and positively associated with food and water safety awareness (P < 0.00). The finding of this study revealed that, 76.0% of the participants with the ability of at least to read and write had a higher level of awareness of food and water safety. This is supported with research outcomes [20],[30]. Similarly, in our study, length of work experience as food handlers was positively and significantly associated (P = 0.02) with awareness. This result was consistent with the study done in Salvador, Brazil [25]. However, in our study, marital status or being married (P < 0.00) was negatively and significantly associated with awareness of food handlers. This may need further study to obtain additional information. Further, the linear regression model analysis indicated that, having awareness on food and water safety (F = 252.49; df = 1; P = 0.00) with Standardized Coefficient (Beta) = 0.62 at 95% CI correlates positively and significantly with having good outlook. This indicated that, having good awareness had significant positive effect on outlook although it needs further studies to obtain additional evidences.

## 5. Conclusion

Assessing awareness, outlook and practice of food handlers regarding food and water safety is a vital activity to reduce public health problems. In this study, food and water safety awareness had a significant effect on food and water safety practices. Significant number of food handlers had poor awareness, outlook and practice towards food and water safety. There is a call for enhancing the awareness, outlook and practice of food and water safety to achieve an excellent practice. Better food and water safety policy and firm regulatory actions are needed.

## 6. Recommendation

Strong food and water safety policy and strategy should be promulgated. In addition, firm regulatory body assisted with law enforcement, guidelines and manuals should be in place to upgrade awareness, outlook and practice of food handlers and make food establishments adhere to the policy, strategy and guidelines. Moreover, Governmental and Non-Governmental organizations should conduct continuous transformational information management system and capacity building through formal training on food and water safety to food handlers and owners of food establishments to reduce food and waterborne diseases in the community.

## 7. Limitation of the study

The main limitation of this study was social desirability bias in the action questions.
